# The age‐related incidence of male genital lichen sclerosus is triphasic

**DOI:** 10.1002/ski2.447

**Published:** 2024-09-05

**Authors:** Cherry Choudhary, Ryan Beazley, Encarl Uppal, Georgios Kravvas, Christopher Bunker

**Affiliations:** ^1^ Department of Dermatology University College London Hospitals London UK; ^2^ Department of Medical Education Brunel Medical School Brunel University London London UK

## Abstract

**Background:**

Male genital lichen sclerosus (MGLSc) is a chronic and acquired inflammatory dermatosis associated with substantial sexual dysfunction and urological morbidity and mortality. The age incidence of MGLSc is held to be biphasic, with a peak in infancy and another in adulthood. A recent review has implied two peaks in adulthood (making it triphasic overall); this triphasicity has been our emergent clinical impression from a voluminous practice. Furthermore, a link between MGLSc and smoking has been suggested, but this has not been our clinical impression.

**Objectives:**

The primary objective was to clarify the age‐specific incidence of adult men with GLSc; the secondary objective was to explore the relationship between MGLSc and smoking.

**Methods:**

We retrospectively reviewed the medical notes of 487 adult MGLSc patients from a large specialist male genital dermatology clinic. We abstracted data about the age of diagnosis and smoking history.

**Results:**

A biphasic U‐shaped age distribution was identified with two clear peaks at the end of the third decade and another in the sixth decade of life (Hartigan's dip‐stat = 0.03; *p* < 0.01). Thirty‐six percent of the patients had been smokers at some point in their lives.

**Conclusions:**

These findings confirm that MGLSc is biphasic in its adult incidence, confirming an earlier supposition; including the previously well‐acknowledged paediatric peak, it is thus triphasic. The smoking data are probably unremarkable compared with the available data for smoking habits from the United Kingdom. These findings indirectly support what is postulated about the likely pathogenesis of MGLSc, that is, urinary micro‐incontinence, occlusion and epithelial susceptibility.

## Graphical abstract

In a retrospective study of 487 patients with male genital lichen sclerosus (MGLSc) at a large specialist male genital dermatology clinic, we confirmed an adult biphasic distribution with peaks in the third and sixth decades. We found no significant association between MGLSc and smoking. Combined with the known paediatric peak, our findings support the triphasic pattern of MGLSc incidence and reflect the suggested pathogenesis involving urinary micro‐incontinence, occlusion and epithelial susceptibility.

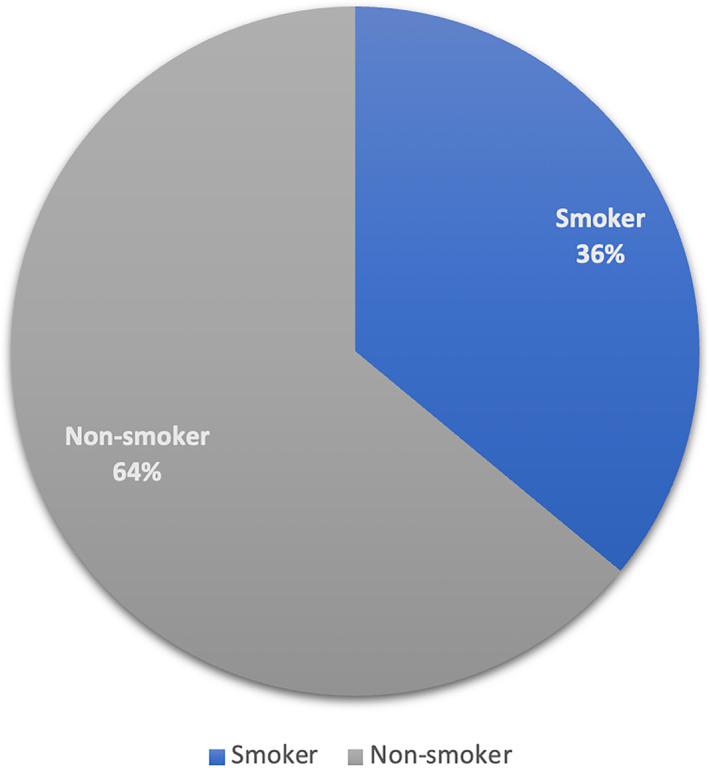

**What is already known about this topic?**

- The existing literature suggests a bimodal age distribution in the incidence of Male genital lichen sclerosus (MGLSc) in childhood and in adulthood. Peaks have previously been observed in adolescence and around the fourth decade of life. However, a more recent review in men postulated that there were data to suggest a peak in the sixties.

**What does this study add?**

- This study reveals a bimodal age distribution of MGLSc in adults. When combined with the acknowledged paediatric age peak, this suggests an overall triphasic age distribution. This evident triphasicity must be accommodated by competing theories for the pathogenesis of MGLSc.

## 1 INTRODUCTION

Male genital lichen sclerosus (MGLSc) is a chronic inflammatory dermatosis that can lead to significant sexual and urological dysfunction.^1^ The precise aetiopathogenesis of MGLSc remains contested. Various causative factors, such as autoimmunity, infection and physical trauma, have previously been suggested, with only limited published evidence available.^2^, ^3^ However, an expanding body of research suggests that chronic exposure of urine may play a central role. Due to post‐void micro‐incontinence and the occlusive property of the prepuce, urine can irritate a susceptible epithelium and cause inflammation and subsequent sclerosis.^4^

Another potential factor that could influence the development of MGLSc is smoking. It is postulated that this could be due to inflammation and microvascular damage. Smoking is a known risk factor for penile cancer; however, the potential link between smoking and MGLSc is tenuous; one study has shown a positive correlation,^5^ whereas another has demonstrated no significant relationship.^6^

Received wisdom is that there is a bimodal age distribution in the incidence of MGLSc in childhood and in the fourth decade of adult life.^2^ However, a further peak in the sixth decade of life had also been suggested.^7^

Clarifying the epidemiology of MGLSc impacts theories about pathogenesis, prevention, diagnosis and management.

## 2 OBJECTIVES

The primary objective of this study was to evaluate the age‐specific incidence of adult MGLSc.

A secondary objective was to investigate smoking behaviours.

## 3 METHODS

### 3.1 Study design

A retrospective analysis of data was performed on adult males presenting with MGLSc between September 2019 and February 2024 to our specialist male genital dermatology and andrology service. All male patients over the age of 18 were included within this analysis. 487 patients were identified and all were included in the analysis. Age at diagnosis and smoking status were abstracted. These data were obtained from electronic medical notes, outpatient letters, histological reports, and multidisciplinary team reports. Comparable data about UK smoking habits were obtained from the Office of National Statistics (ONS).^8^

### 3.2 Data analysis

Statistical significance of the age peaks obtained was performed by assessing for multimodality using Hartigan's dip test. A confidence level of 99% was used with a *p* value of 0.01. The dip test evaluates the presence of multimodality within a dataset by identifying the greatest difference across all points of data between the empirical distribution function and the closest unimodal distribution function.^9^, ^10^ Data within the peaks were assessed by calculating standard descriptive statistics and the interquartile range. All analyses were performed using Python 3.11.7.

## 4 RESULTS

**FIGURE 1:**
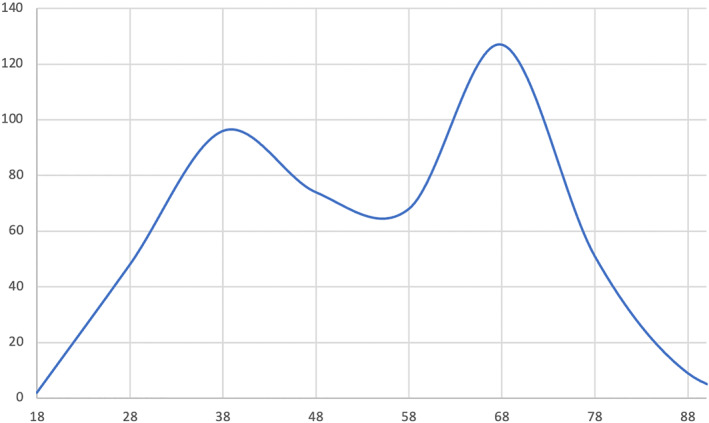
The age distribution of patients diagnosed with MGLSc. A U‐shaped biphasic peak can be observed around the ages of 30–40 and 60–70 years, respectively. MGLSc, Male genital lichen sclerosus.

Between September 2019 and February 2024, 487 adult males were identified. A biphasic U‐shaped age distribution was found, with two clear peaks (Figure 1). The first peak was found in the third decade (mean 39.91, median 39, mode 37, and SD 5.45). The interquartile range for this peak was 9. A second peak was observed in the sixth decade of life (mean 64.86, median 64, mode 62, and SD 4.97). The interquartile range for this peak was 7. As the data did not manifest a normal distribution, conventional analyses could not be used. To assess for age peaks within the data, multimodality was utilised. Hartigan's dip test was used to determine statistical significance. A dip‐stat value of 0.03 (*p* < 0.01) was obtained, showing statistically significant bimodality within the data, confirming the graphical impression.

**FIGURE 2:**
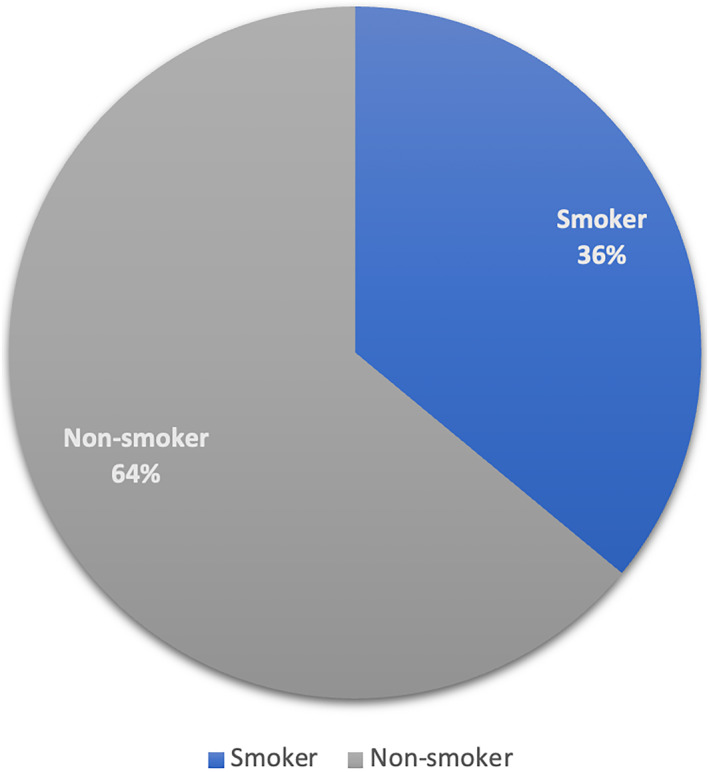
The percentage of current/previous smokers and non‐smokers in those with a diagnosis of MGLSc. MGLSc, Male genital lichen sclerosus.

Out of all patients within our sample, 175 (35.9%) were current or former smokers (Figure 2). According to the Office National Statistics (ONS), the UK adult male incidence of either a current or former smoker is 40.1%.^7^ As such, if this were extended to our sample, we could expect 196 smokers. Compared with our data, there does not appear to be a difference between the incidence of smoking in the MGLSc population and the general male population.

## 5 DISCUSSION

Historically, the literature has alluded to a bimodal age distribution for MGLSc, pre‐pubertally and later in the fourth decade. Our retrospective study on 487 patients clearly demonstrates that there are two peaks in adult life. Therefore, including the acknowledged peak in childhood, the age incidence of MGLSc is actually triphasic.

The exact incidence of MGLSc within the male population is likely underrepresented due to the variability of symptoms and signs and misdiagnosis.^7^ Nonetheless, the three peaks reflect our own emergent clinical impression of many years in specialist genital practice and a closer appraisal of the literature.

Data initially published by Kizer et al., from a relatively small study sample, hinted at the presence of a biphasic age distribution in adults, although these authors did not themselves make that statement.^11^ This was a retrospective observation by De Luca et al. who appear to have adduced a second peak in the seventh decade of life from Kizer's original data.^6^ Similarly, the data of Hieta et al. hints at the presence of a biphasic adult distribution in their MGLSc cohort; however, they also did not specifically comment on this.^12^ The male data of Virgili et al. do suggest increased incidence rates after the sixth decade of life; however, again this was not explicitly analysed nor commented on.^13^ Nelson and Peterson reported an age‐related increase in cases from the third decade onwards, peaking in ages above 60; although their paper confuses incidence and prevalence, it is probably incidence rates that they were reporting.^14^ Data from Sweden suggest peaks in children and very early adulthood, but flattish incidences thereafter.^15^, ^16^, ^17^

Age‐specific incidence data may be useful in several ways namely, illuminating aetiopathogeneses and impacting prevention, screening, diagnosis, and management. In this study, the third peak in the sixth decade of life is higher than the second peak in the third decade. This could be due to differences in healthcare accessibility in different age groups or because of the circumcision of younger males for ‘physiological phimosis' without specifically diagnosing MGLSc. It is also conceivable that the diagnosis of LSc in older age groups is more refined in our institution because of our highly specialist configuration. These are topics of ongoing enquiry by our group.

The definitive pathogenesis of MGLSc has not been universally agreed, but the body of evidence for urinary occlusion of a susceptible epithelium is larger than that for autoimmunity or infection.^16^, ^17^, ^18^ Whilst this paper will not rehearse the urinary hypothesis in detail, central to it is the observation that most men with GLSc ‘dribble’ due to micro‐incontinence.^3^ This may be congenital or acquired. Those with *forme fruste* or actual frank hypospadias could account in part for the childhood peak.^19^, ^20^, ^21^ As would, also, those with an innately more susceptible epithelium to urinary occlusion. In early adulthood, the ‘tipping point’ might be occasioned by the onset of sexual activity and exposure to sexually transmitted and other infections. In later life, increasing or new urinary micro‐incontinence and/or the epithelial susceptibility might be speculatively related to ageing tissue, change of partners (divorce and widowhood), urological procedures,^3^ diabetes, and obesity.^4^ However, it is challenging, even speculatively, to accommodate and explain the three peaks of incidence by autoimmune and infectious hypotheses.

Smoking has been postulated as a potential aggravator and pathogenic factor in MGLSc. In conditions such as lichen planus, nicotine and C‐Met upregulation in smokers have been found to exacerbate symptoms and contribute to inflammation and immune dysregulation.^20^, ^22^ This could also be the case for MGLSc. Hofer et al. found a 107% increase in the incidence of smoking between stricture patients who had MGLSc and those who did not.^5^ In contrast to this, Bjekic et al. found no significant difference in MGLSc incidence between current or former smokers and non‐smokers in their case–control study.^6^ Within our data, only 36% (175) of adult male patients reported smoking at some point in their life. The adult male incidence of either a current or former smoker is 40.1%. This is an average of the entire UK male population, with a different age distribution from the age distribution of LSc patients. Smoking census data from 2021 show that different age groups have different smoking habits. Due to the difference in age distribution from this national data and our LSc data set, direct comparisons of smoking numbers cannot be made, but the implication is that smoking does not appear to be strikingly associated with MGLSc.^8^ However, this may not be a true representation of the smoking statistics for our data set as smoking history was at times poorly or incompletely recorded. At the present time, we conclude that smoking does not appear to be associated with MGLSc, but remains an established risk factor for penis cancer, as indeed MGLSc is itself.

These findings clearly demonstrate, for the first time, the biphasic adult incidence of MGLSc, validating an earlier supposition and revealing a triphasic pattern when combined with the well‐established paediatric peak. No clear link between smoking and MGLSc emerges from our study. Additionally, these data may align best with the now dominant theory about the pathogenesis of MGLSc, *viz*. occlusion of urine (due to micro‐incontinence) and epithelial susceptibility. We plan to scrutinise a larger cohort of patients for more accurate information about smoking.

## CONFLICT OF INTEREST STATEMENT

Cherry Choudhary is an Associate Editor for Skin Health and Disease.

## AUTHOR CONTRIBUTIONS

**Cherry Choudhary**: Data curation (lead); formal analysis (supporting); investigation (equal); methodology (equal); project administration (lead); resources (lead); visualization (supporting); writing – original draft (lead); writing – review & editing (lead). **Ryan Beazley**: Formal analysis (lead); project administration (supporting); software (lead); validation (lead); visualization (lead); writing – original draft (supporting). **Encarl Uppal**: Writing – original draft (supporting). **Georgios Kravvas**: Conceptualization (supporting); investigation (equal); methodology (equal); supervision (supporting). **Christopher Bunker**: Conceptualization (lead); data curation (supporting); investigation (equal); methodology (equal); supervision (lead); writing – original draft (supporting); writing – review & editing (supporting).

## ETHICS STATEMENT

Not applicable.

## PATIENT CONSENT

Not applicable.

## Data Availability

The data that support the findings of this study are available on request from the corresponding author. The data are not publicly available due to privacy or ethical restrictions.
